# Supplementary material for: Measurement of the variation of electron-to-proton mass ratio using ultracold molecules produced from laser-cooled atoms
Source: Nat Commun. 2019 Aug 21;10:3771. doi: 10.1038/s41467-019-11761-1 (PMC6704166; doi:10.1038/s41467-019-11761-1)
Supplement: Supplementary file 1 — Supplementary Information [file 41467_2019_11761_MOESM1_ESM.pdf]

Supplementary Information for "Measurement of the variation of electron-to-proton mass ratio using ultracold molecules produced from laser-cooled atoms"

Kobayashi et al.

Supplementary note 1

As given in Supplementary table 1, we observed four singlet and six triplet states by STIRAP spectroscopy. The hyperfine structures were analyzed based on the Hamiltonian

$$H = A_{\mathrm{K}}\mathbf{S}_{\mathrm{K}} \cdot \mathbf{I}_{\mathrm{K}} + A_{\mathrm{Rb}}\mathbf{S}_{\mathrm{Rb}} \cdot \mathbf{I}_{\mathrm{Rb}}, \tag{1}$$

where $\mathbf{S}_{\mathrm{K}}(\mathbf{S}_{\mathrm{Rb}})$ and $A_{\mathrm{K}}(A_{\mathrm{Rb}})$ are the electron spin and hyperfine coupling constant for the K(Rb) atom, respectively. The fitting parameters are the energy separation (E_0) between $|X^1\Sigma^+, v = 86, N = 0\rangle$ and $|a^3\Sigma^+, v = 16, N = 0\rangle$ states without the hyperfine interaction and hyperfine coupling constants (A_{K} and A_{Rb}) for both atoms. In the analysis, we ignore mixing with higher rotational states because it is less important. The fitting agrees well with the experimental data as shown in Supplementary table 1. From the analysis, we can predict the singlet-triplet mixing, the Zeeman coefficients, and the sensitivity to the variation of μ as shown in Supplementary table 1.

	F_1	F	$E_{\rm exp}$ (MHz)	$E_{\rm cal}$ (MHz)	ρ_X (%)	$\epsilon_{\rm B}$ (kHz μT^{-2})	W (GHz)
$a^3\Sigma^+$, $v=16$, $S=1$	5/2	4	-	7448.16	0.0000	0.693	1783
		3	-	7349.17	0.0009	0.880	1783
		2	-	7273.68	0.0008	1.128	1783
		1	-	7222.73	0.0004	-2.475	1783
	3/2	3	3231.81	3231.87	1.93	0.558	1970
		2	3190.73	3190.76	2.09	0.961	1985
		1	3160.29	3160.28	2.22	3.239	1997
		0	3144.01	3143.94	2.29	-4.649	2004
	**1/2**	**1**	**634.94**	**634.87**	**0.113**	**0.806**	**1794**
		2	550.20	550.26	0.254	-1.108	1807
$X^1\Sigma^+$, $v=86$, $S=0$	**3/2**	3	12.29	12.30	98.07	-0.0084	11283
		2	5.10	5.14	97.65	-0.0076	11242
		1	1.54	1.56	97.67	-0.0022	11243
		**0**	**0**	**0**	**97.71**	**-0.0164**	**11248**

Supplementary table 1: Hyperfine states of the $|X^1\Sigma^+, v=86\rangle$ and $|a^3\Sigma^+, v=16\rangle$ states. The hyperfine states are denoted as $|S, F_1, F, m_F\rangle$. $E_{\rm exp}$ and $E_{\rm cal}$ are energies obtained from the experiment and the data fitting, respectively. Both are measured from the $|S=0, F_1=3/2, F=0\rangle$ state. Singlet components (ρ_X), second order Zeeman shift coefficients for $m_F=0$ states($\epsilon_{\rm B}$), and the sensitivity to the variation of the electron-to-proton mass ratio ($W \equiv \partial E/\partial(\ln\mu)$) are also shown. The obtained value for E_0 (4720.27 MHz) is consistent with the predicted value (4178.75 MHz) in Ref.[1]. The obtained values for $A_{\rm K}$ (125.54 MHz) and $A_{\rm Rb}$ (3384.99 MHz) are slightly smaller than the hyperfine coupling constants for bare atoms(*i.e.*,127.007 MHz and 3417.341 MHz, respectively [2]) and result from the dependence of the coupling constants on the internuclear distance. The transition between $|0, 3/2, 0, 0\rangle$ and $|1, 1/2, 1, 0\rangle$ states, indicated by boldface type, was used to test the stability of μ.

[1] Pashov, A. *et al.* Coupling of the $X^1\Sigma^+$ and $a^3\Sigma^+$ states of KRb. *Phys. Rev. A* **76**, 022511 (2007).
[2] Arimondo, E., Inguscio, M. & Violino, P. Experimental determinations of the hyperfine structure in the alkali atoms *Rev. Mod. Phys.* **49**, 31-75 (1977).
